# Massed *v*. standard prolonged exposure therapy for PTSD in military personnel and veterans: 12-month follow-up of a non-inferiority randomised controlled trial

**DOI:** 10.1017/S0033291723000405

**Published:** 2023-11

**Authors:** Lisa Dell, Alyssa M. Sbisa, Andrew Forbes, Meaghan O'Donnell, Richard Bryant, Stephanie Hodson, David Morton, Malcolm Battersby, Peter W. Tuerk, Peter Elliott, Duncan Wallace, David Forbes

**Affiliations:** 1 Phoenix Australia – Centre for Posttraumatic Mental Health, Department of Psychiatry, The University of Melbourne, Melbourne, Victoria, Australia; 2 School of Public Health and Preventive Medicine, Monash University, Melbourne, Victoria, Australia; 3 School of Psychology, University of New South Wales, Sydney, New South Wales, Australia; 4 Department of Veteran's Affairs, Canberra, Australian Capital Territory, Australia; 5 Defence, Canberra, Australian Capital Territory, Australia; 6 College of Medicine and Public Health, Flinders University, South Australia, Australia; 7 Department of Human Services, Sheila C. Jonson Center for Clinical Services, University of Virginia, Charlottesville, Virginia, USA; 8 Australian Defence Force Centre for Mental Health, Sydney, New South Wales, Australia

**Keywords:** Massed therapy, military, non-inferiority, prolonged exposure therapy, PTSD, RCT, trauma, veteran

## Abstract

**Background:**

The utilisation of massed therapy for treating posttraumatic stress disorder (PTSD) is gaining strength, especially prolonged exposure. However, it is unknown whether massed prolonged exposure (MPE) is non-inferior to standard prolonged exposure (SPE) protocols in the long term. The current study aimed to assess whether MPE was non-inferior to SPE at 12 months post-treatment, and to ascertain changes in secondary measure outcomes.

**Methods:**

A multi-site non-inferiority randomised controlled trial (RCT) compared SPE with MPE in 12 clinics. The primary outcome was PTSD symptom severity (CAPS-5) at 12 months post-treatment commencement. Secondary outcome measures included symptoms of depression, anxiety, anger, disability, and quality of life at 12 weeks and 12 months post-treatment commencement. Outcome assessors were blinded to treatment allocation. The intention-to-treat sample included 138 Australian military members and veterans and data were analysed for 134 participants (SPE = 71, MPE = 63).

**Results:**

Reductions in PTSD severity were maintained at 12 months and MPE remained non-inferior to SPE. Both treatment groups experienced a reduction in depression, anxiety, anger, and improvements in quality of life at 12 weeks and 12 months post-treatment commencement. Treatment effects for self-reported disability in the SPE group at 12 weeks were not maintained, with neither group registering significant effects at 12 months.

**Conclusions:**

The emergence of massed protocols for PTSD is an important advancement. The current study provides RCT evidence for the longevity of MPE treatment gains at 12 months post-treatment commencement and demonstrated non-inferiority to SPE. Promisingly, both treatments also significantly reduced the severity of comorbid symptoms commonly occurring alongside PTSD.

## Introduction

Although there exists well-established evidence-based treatments for posttraumatic stress disorder (PTSD) (Kline, Cooper, Rytwinksi, & Feeny, 2018; Lewis, Roberts, Andrew, Starling, & Bisson, 2020), a recent focus of researchers has included improving outcomes by testing different intensities of delivery. This is of particular importance as it has been hypothesised delivering treatment intensively may increase compliance and reduce rates of dropout (Levinson, Halverson, Wilson, & Fu, 2022). Indeed, while standard prolonged exposure (SPE) is considered a first-line treatment for PTSD, dropout rates of nearly 40% have been reported (Kehle-Forbes, Meis, Spoont, & Polusny, 2016). Recently, we reported a shorter yet more intensive course of prolonged exposure therapy (massed prolonged exposure; MPE), occurring daily over the course of 2 weeks rather than once-weekly over 10 weeks (SPE), in military personnel and veterans showed MPE was non-inferior to SPE in reducing PTSD severity, with the additional benefit of reduced dropout (Dell et al., 2022).

As evidence for the immediate benefits of massed treatment builds, including virtually via telehealth (Held et al., 2021, 2022), a critical question is whether treatment gains from a shorter term and more intensive therapy are maintained in the long term to the same extent as a standard course of treatment (Held et al., 2020), particularly given the shorter duration for the consolidation of new associations. Inhibitory learning theory, which exposure therapy is grounded upon, posits the association learned during fear acquisition is not erased nor replaced, but rather a new association (conditioned stimulus without the unconditioned stimulus) becomes the dominant response (Tolin, 2019). It has been suggested that inhibitory learning is enhanced and better maintained when employing a spaced or expanding-spaced schedule, rather than massed (Craske, Treanor, Conway, Zbozinek, & Vervliet, 2014; Tolin, 2019; Tsao & Craske, 2000). A recent study using at 6-month follow-up found MPE treatment outcomes were maintained at 6 months following therapy (Foa et al., 2018), which is promising, but the question of longer term maintenance remains unanswered. This randomised controlled trial (RCT) is the first multi-centre trial examining MPE outcomes at 12 months following treatment commencement and represents a key contribution to our understanding of the utility of modified first-line therapies.

Alongside understanding whether treatment gains are maintained, a question exists on the impact of massed first-line treatments on comorbid mental health issues, a common occurrence in PTSD in military personnel and veterans. Although the primary aim of prolonged exposure is to address trauma-related memories, some studies have reported the amelioration of depressive symptoms (Aderka, Foa, Applebaum, Shafran, & Gilboa-Schechtman, 2011; Eftekhari et al., 2013; Nacasch et al., 2010), general anxiety (van Minnen, Zoellner, Harned, & Mills, 2015), and anger (Ford, Grasso, Greene, Slivinsky, & DeViva, 2018). More recently, the evidence is building for similar effects of MPE on anxiety and depression (Hendriks et al., 2017; Zwetzig, Koch, Blount, Graham, & Peterson, 2022); however, the impact on anger severity is unknown. Further, whether MPE demonstrates comparable reductions and longevity in comorbid mental health symptoms to SPE, given a briefer duration of time for these issues to manifest and consequently be addressed within treatment, is yet to be determined.

The aim of this paper was to extend the previously published 12-week outcomes (Dell et al., 2022) and examine whether the longevity of treatment gains for MPE was non-inferior to SPE at 12 months post-treatment commencement, and to ascertain whether MPE was non-inferior to SPE with regards to impacts on secondary outcome measures. It was hypothesised that MPE gains would be maintained and remain non-inferior to SPE treatment at 12 months, and MPE would be non-inferior to SPE in reducing the severity of depression, anxiety, anger, disability, and improving the quality of life at 12 weeks, and maintained at 12 months.

## Methods

For a detailed description of the methodology see the trial protocol (Dell et al., 2021) and initial primary outcome findings (Dell et al., 2022). Briefly, this multi-site non-inferiority RCT took place in 12 health clinics across eight sites within eight states and territories in Australia, and via telehealth during the COVID-19 pandemic. While the trial protocol was originally designed for face-to-face (F2F) therapy, this was adapted to include telehealth during the pandemic and Australian lockdown periods given evidence that telehealth therapy, including prolonged exposure, provides equivalent outcomes to F2F therapy for patients with PTSD (Scott et al., 2022).

Participants underwent a pre-treatment baseline assessment (T1) including a clinical interview and a self-report booklet. Immediately following T1 assessment, eligible participants were randomly allocated in a 1:1 ratio to SPE or MPE therapy. Randomisation occurred using two components – randomisation to SPE or MPE, and then randomisation to a therapist to deliver the intervention. Assessors (blinded to condition) conducted follow-up assessments at 4 weeks post-treatment commencement (T2), 12 weeks post-treatment commencement (T3), and 12 months post-treatment commencement (T4).

The Australian Defence Human Research Ethics Committee and Department of Veterans' Affairs Human Research Ethics Committee approved the protocol (now known as the Departments of Defence and Veterans' Affairs Human Research Ethics Committee), see Dell et al. (2021). The authors assert that all procedures contributing to this work comply with the ethical standards of the relevant national and institutional committees on human experimentation and with the Helsinki Declaration of 1975, as revised in 2008.

### Participants

Inclusion criteria were: aged 18–80 years, current or former Australian Defence Force member, PTSD diagnosis as determined by the Clinician-Administered PTSD Scale for DSM-5 (CAPS-5), and a Criterion A trauma that occurred while serving. Exclusion criteria included current: clinician-rated psychosis, mania, high risk of harm to self or others, and severe alcohol or substance-use disorder as determined by the Mini International Neuropsychiatric Interview (MINI), and receiving other trauma-focused psychological therapy and unwilling/unable to pause. Given the prominence of risk within this population, risk to self and others was assessed at several points through the trial, including the initial intake call, T1 baseline assessment, and where needed during therapy. High risk was defined as active thoughts to harm self or others and the potential for immediate harm, or recent risk with the potential to be exacerbated by trauma-focused therapy. Risk to self or others was rated as no foreseeable, low, medium, or high, and previous suicidality did not preclude individuals from participation in therapy. Individuals taking psychotropic medication were required to be on a stable dose (no titration) for at least 4 weeks prior to commencing and without intention to change for the duration of treatment. All participants provided informed written consent to participate in the trial.

### Treatment intervention

The SPE group received 10 weekly sessions of 90-min F2F manualised therapy (Foa, Hembree, & Rothbaum, 2007) and the MPE group received identical therapy delivered rapidly over 2 weeks (Dell et al., 2021). Both groups undertook *in vivo* homework activities. Telehealth treatment (offered during the Australian COVID-19 restrictions) involved the same format and duration of therapy, however took place using online video platforms. Treatment completers were defined by attendance at a minimum of seven sessions, consistent with previous prolonged exposure research (Sripada & Rauch, 2015; Tuerk et al., 2013; Yoder, Tuerk, & Acierno, 2010). Scheduled therapy sessions impeded by personal circumstances, such as illness, were rescheduled, and for a small minority, MPE participants completed therapy in <3 weeks and SPE within 12 weeks.

For both groups, therapists contacted the participant over phone 1-, 3-, and 6-weeks post-therapy to encourage the participant to continue undertaking *in vivo* activities and to monitor activity, as is a common practice in disseminated settings. Treatment fidelity was conducted by an independent expert listening to and grading audio recordings of every session for a therapist's first participant, for session one and three of the therapist's second participant, and then on a random sample of 10% of all cases. In addition, therapists attended fortnightly supervision to ensure adherence to protocol. Monthly clinical supervision was also provided for assessors to ensure CAPS-5 inter-rater reliability.

### Outcome measures

#### CAPS-5

Posttraumatic stress symptom severity at 12 months (T4) was measured by the clinician-rated measure for PTSD, the CAPS-5 (Weathers et al., 2018). The structured clinical interview is comprised of 30-items scored on a 5-point Likert scale measuring symptoms clusters of avoidance, negative alterations in cognition and mood, arousal and reactivity, and re-experiencing during the past month. The CAPS-5 provides an overall severity score ranging from 0 to 80, with moderate scores ranging from 23 to 34, severe scores between 35–47, and extreme ⩾48. The CAPS-5 is one of the most widely used tools for diagnosing and measuring PTSD severity, with excellent reliability and validity (Weathers et al., 2018). PTSD symptoms were assessed for the past month at T1 and T4 and assessed for the past 2 weeks at T2 and T3.

#### DAR-5

Problematic anger was measured by the Dimensions of Anger Reactions-5 (DAR-5) self-report questionnaire (Forbes et al., 2014b) at all time points. The DAR-5 is a 5-item measure of anger frequency, intensity, duration, aggression, and impact on relationships, with individuals responding on a scale of 0 (none or almost none of the time) to 5 (all or almost all of the time). The DAR-5 demonstrates strong internal reliability, convergent concurrent and discriminant validity, and, particularly relevant to this study, has been validated in populations with a trauma history (Forbes et al., 2014b), and with combat veterans (Forbes et al., 2014a).

#### HADS anxiety and depression

The Hospital Anxiety and Depression Scale (HADS) (Zigmond & Snaith, 1983) was used to measure anxiety and depression symptoms at all time points. The HADS is a 14-item self-report measure of depression and anxiety symptoms, asking respondents to indicate from 0 to 3 the frequency or intensity with which they experience each symptom. A score of 8 on either the depression or anxiety subscales indicates pathology (Bjelland, Dahl, Haug, & Neckelmann, 2002). Review of the psychometric properties of the HADS, incorporating over 700 studies, indicated satisfactory sensitivity, specificity, and concurrent validity for both anxiety and depression subscales for those with physical or mental health difficulties and the general population (Bjelland et al., 2002).

#### WHODAS

Disability was assessed at all time points by the 12-item self-report version of the World Health Organization Disability Assessment Schedule 2.0 (WHODAS 2.0) (Ustun et al., 2010). The WHODAS 2.0 asks individuals to indicate their level of difficulty due to health conditions in the areas of understanding and communication, self-care, mobility, interpersonal relationships, work and household activities, and community roles (Marx et al., 2015; Ustun et al., 2010). The WHODAS 2.0 demonstrates good internal consistency, reliability, and concurrent validity in a wide range of populations and cultures (Marx et al., 2015; Ustun et al., 2010). Specifically relevant to this study, the interview version of the WHODAS 2.0 has been validated as an assessment of functional impairment among veterans, identifying those with PTSD-related impairment assessed by the CAPS-5 (Marx et al., 2015).

#### AQoL-6D

Quality of life was assessed at each time point by the Assessment of Quality of Life Scale – 6 dimension version (AQoL-6D) (Maxwell, Özmen, Iezzi, & Richardson, 2016). The AQoL-6D is a 20-item self-report questionnaire reflecting physical and psychosocial domains. Respondents indicate their level of functioning in the areas of independent living, relationships, mental health, coping, pain, and senses (Allen, Inder, Lewin, Attia, & Kelly, 2013). In a large Australian sample, it demonstrated satisfactory levels of construct, concurrent, and convergent validity (Allen et al., 2013). Total scores range from 1.00 (full health) to 0.00 (death-equivalent health states) to −0.04 (health states worse than death) (Hawthorne & Osborne, 2005), a positive score on the AQoL-6D indicates an improvement in quality of life.

### Statistical analysis

Baseline characteristics of the MPE and SPE groups were compared using descriptive statistics. Number of participants and group percentages are presented for categorical data, with means and standard deviations presented for continuous data. Treatment groups were compared across a number of demographic and service characteristics, and on their baseline scores on the primary and secondary outcome measures.

Differences between groups and across time in the scores on the outcome measures were examined using linear mixed-effects models (LMMs). The LMM procedure accommodates both correlated data and unequal variances, and includes both between groups and repeated measures (within-groups) effects at the same time. Participants' scores are deemed to be the result of both fixed effects (e.g. treatment group) and random (e.g. subject) effects, and LMM utilises maximum-likelihood and restricted maximum-likelihood methods to yield optimum estimators (with minimum variance) for balanced and unbalanced designs. Separate LMMs were conducted for each outcome measure. For all measures except for the AQoL-6D, a negative score indicates a reduction in symptoms. For the AQoL-6D a positive score indicates an improvement in quality of life. While not included in the original protocol (Dell et al., 2021), but given it is routinely reported, prior to any data analysis there was a decision to assess for non-inferiority in loss of diagnosis.

Multiple imputation of missing CAPS-5 data at T2 (MPE 15.9%; SPE 26.8%), T3 (MPE 22.2%; SPE 25.4%), and T4 (MPE: 39.7%; SPE: 42.3%) was performed using SPSS with variables in the imputation model chosen if they correlated strongly with CAPS-5 diagnoses being imputed. Imputations were conducted separately for each treatment group to produce 10 imputed datasets. The pooled estimates of the frequencies of CAPS-5 diagnoses were then subjected to χ^2^ tests for differences between groups at each of the time points, and to McNemar's tests for differences across time within groups.

PTSD diagnostic status was determined by dichotomising individual symptoms as ‘present’ or ‘absent’, following the DSM-5 diagnostic algorithm. A symptom was considered present only if the corresponding item severity score was rated 2 (moderate/threshold) or higher. In the CAPS-5, items 9 and 11–20 have the additional requirement of a trauma-relatedness rating of definite or probable, otherwise a symptom is considered absent. The DSM-5 diagnostic rule requires the presence of at least one Criterion B symptom, one Criterion C symptom, two Criterion D symptoms, and two Criterion E symptoms. In addition, Criteria F and G must be met. Criterion F requires that the disturbance has lasted at least 1 month. Criterion G requires that the disturbance causes either clinically significant distress or functional impairment, as indicated by a rating of 2 (moderate) or higher on items 23–25. This calculation of diagnosis is consistent with Voorendonk, De Jongh, Rozendaal, and Van Minnen (2020).

Power and sample size were based on the upper endpoint of the 95% confidence interval (CI) for the difference in mean change scores from baseline to 12 weeks between MPE and SPE (adjusted for baseline) being less than the margin of 7 CAPS-5 points, the outcomes of which have been reported elsewhere (Dell et al., 2022). See Dell et al. (2022) for detailed information regarding sample size calculation.

All analyses utilised SPSS v27. A *p* value of <0.05 was considered significant. Cohen's effect sizes for repeated measures are also presented, and small, medium, and large effect sizes correspond to 0.2, 0.5, and 0.8, respectively.

## Results

**Fig. 1.**
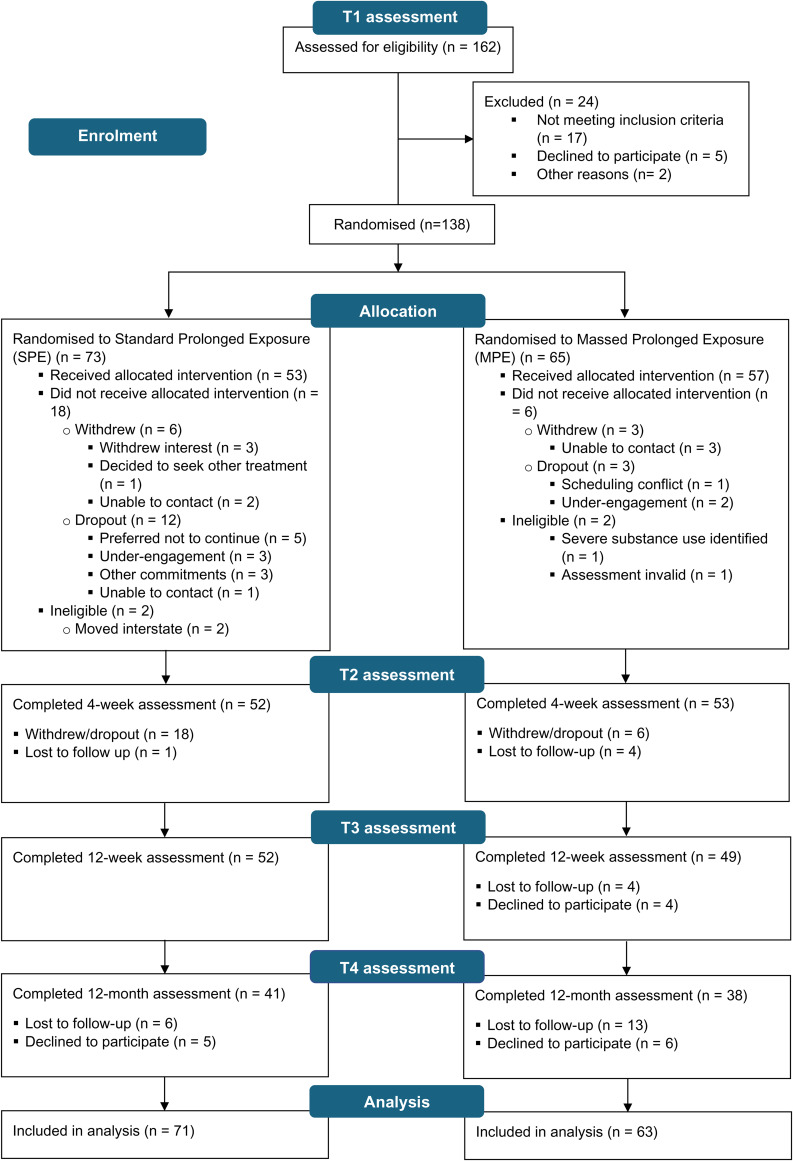
CONSORT diagram describing flow of participants through the study. *Notes.* CONSORT, Consolidated Standards of Reporting Trials. Withdrew, participants who were randomised to a condition but did not commence treatment. Dropout, participants who were randomised to a condition, commenced treatment but discontinued. Ineligible, ineligible and excluded from analysis. Participants who withdrew or dropped out of therapy were not invited to undergo follow-up assessments.

One hundred and sixty-two individuals were assessed for eligibility at T1, and 138 current and ex-serving Australian Defence Force (ADF) members with PTSD were randomised to therapy in the RESTORE trial. Of the 138 who were found to be eligible and randomised to therapy, four were later found to be ineligible and were excluded (see CONSORT diagram in Fig. 1). The final intention-to-treat sample comprised of 134 participants who were randomised to MPE (*n* = 63) or SPE (*n* = 71).

Demographic and service characteristics for the sample were collected via participant self-report during the baseline T1 assessment, as reported in Dell et al. (2022), and showed no significant differences between MPE and SPE groups. Thirty-three per cent of both the SPE and MPE groups were current serving ADF members. Baseline mean scores for all measures by treatment group are presented in Table 1. There were no significant differences between the groups on five of the secondary measures. MPE group members reported a significantly higher level of depression on the HADS at baseline than did SPE group members.

**Table 1.** Mean total scores on outcome measures at baseline by treatment group

	MPE (*n* = 63)		SPE (*n* = 71)		
	*M*	s.d.	*M*	s.d.	*p*
CAPS-5	42.38	9.18	39.44	10.43	ns
DAR-5	13.03	4.60	12.90	4.48	ns
HADS anxiety	12.53	3.58	11.94	3.89	ns
HADS depression	11.92	4.01	10.55	3.56	0.040
WHODAS	18.95	7.39	19.99	8.22	ns
AQoL-6D	0.46	0.17	0.46	0.17	ns

AQoL-6D, Assessment of Quality of Life 6D; CAPS-5, Clinician-Administered PTSD Scale for DSM-5; DAR-5, Dimensions of Anger Reactions Scale; HADS, Hospital Anxiety and Depression Scale; MPE, massed prolonged exposure; SPE, standard prolonged exposure; WHODAS, World Health Organization Disability Assessment Schedule 2.0.

**Fig. 2.**
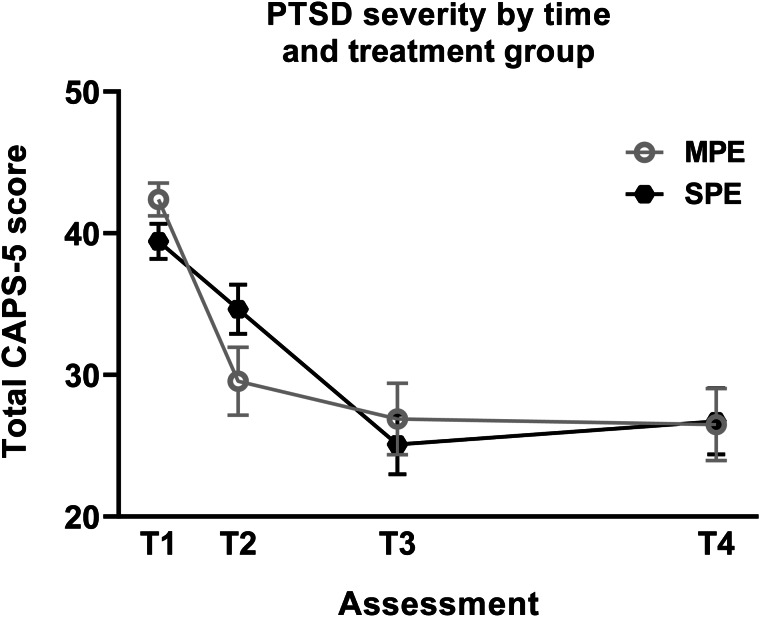
CAPS-5 total score change from baseline (T1), 4-weeks (T2), 12-weeks (T3), and 12 months (T4) post-commencement of therapy. MPE, massed prolonged exposure; SPE, standard prolonged exposure.

At T4, MPE remained non-inferior to SPE. From baseline [MPE (*M* = 42.38, s.d. = 9.18) and SPE (*M* = 39.44, s.d. = 10.43)] to 12 months post-treatment [MPE (*M* = 26.50, s.d. = 15.72) and SPE (*M* = 26.75, s.d. = 14.79)], treatment gains were maintained in both groups, with a 95% CI of −6.82 to +3.92. The upper endpoint of the 95% CI (+3.92) was below +7, indicating non-inferiority. The baseline (T1), and 4 weeks (T2), 12 weeks (T3), and 12 months (T4) post-commencement of therapy scores for the PTSD outcome measure is presented in Fig. 2 with change scores presented in Table 2. From baseline to 12 months (T1 *v.* T4), large effect sizes were observed for change scores within both groups on the CAPS-5 (Table 3).

**Table 2.** Change in scores of outcome measures over time within treatment groups

	T1 *v.* T3	T3 *v*. T4	T1 *v*. T4
Measure	Difference (ES); s.e.; 95% CI	Difference (ES); s.e.; 95% CI	Difference (ES); s.e.; 95% CI
CAPS-5			
MPE	−14.12 (0.98); 1.92; −10.31 to −17.92	0.45 (0.00); 1.69; −2.93 to 3.83	−13.67 (1.00); 1.92; −9.86 to −17.48
SPE	−13.75 (1.05); 1.89; −10.00 to −17.49	1.97 (0.24); 1.65; −1.32 to 5.27	−11.77 (1.01); 1.88; −8.04 to −15.50
DAR-5			
MPE	−1.74 (0.42); 0.70; −0.35 to −3.12	−0.02 (0.00); 0.77; −1.55 to 1.51	−1.76 (0.37); 0.81; −0.14 to −3.37
SPE	−2.50 (0.49); 0.64; −1.23 to −3.77	0.68 (0.22); 0.70; −0.72 to 2.09	−1.82 (0.16); 0.79; −0.25 to −3.38
HADS anxiety			
MPE	−2.29 (0.45); 0.64; −1.01 to −3.56	−0.23 (0.12); 0.60; −1.43 to 0.96	−2.52 (0.61); 0.57; −1.39 to −3.66
SPE	−2.55 (0.76); 0.59; −1.38 to −3.73	−0.08 (0.04); 0.53; −1.14 to 0.98	−2.63 (0.67); 0.55; −1.53 to −3.73
HADS depression			
MPE	−2.50 (0.51); 0.68; −1.14 to −3.86	−0.01 (0.05); 0.57; −1.14 to 1.12	−2.51 (0.63); 0.67; −1.18 to −3.84
SPE	−2.28 (0.58); 0.65; −0.99 to −3.56	0.02 (0.05); 0.49; −0.97 to 1.01	−2.26 (0.64); 0.64; −0.97 to −3.54
WHODAS			
MPE	−0.64 (0.18); 1.14; −2.91 to 1.62	−1.09 (0.25); 1.17; −3.41 to 1.24	−1.73 (0.24); 1.23; −4.18 to 0.71
SPE	−2.61 (0.41); 1.05; −0.52 to −4.70	0.87 (0.16); 1.08; −1.29 to 3.03	−1.74 (0.39); 1.19; −4.12 to 0.63
AQoL-6D			
MPE	0.07 (0.45); 0.03; 0.01–0.12	−0.01 (0.02); 0.03; −0.07 to 0.04	0.05 (0.41); 0.03; 0.00–0.11
SPE	0.07 (0.42); 0.02; 0.02–0.12	−0.01(0.05); 0.03; −0.06 to 0.04	0.06 (0.34); 0.03; 0.01–0.11

*Note.* For all measures except for the AQoL-6D, a negative score indicates a reduction in symptoms.

T1, pre-treatment baseline; T2, 4 weeks post-commencement of therapy; T3, 12 weeks post-commencement of therapy; T4, 12 months post-commencement of therapy; ES, effect size; s.e., standard error.

**Table 3.** Comparison of within-group CAPS-5 changes over specific time periods

						95% CIs	
Period	Estimate	s.e.	df	*t*	*p*	Lower	Upper
T1–T2	7.633	2.641	276	2.891	0.004	2.435	12.832
T1–T3	−0.095	2.722	276	0.035	0.972	−5.455	5.265
T1–T4	1.451	2.705	276	0.536	0.592	−3.873	6.775
T3–T4	1.545	2.362	276	0.654	0.514	−3.105	6.196

*Note*. A positive parameter estimate means that the CAPS-5 change in the MPE group was greater than that for the SPE group.

The proportions of participants with a diagnosis of PTSD on the CAPS-5 was compared across time separately for the MPE and SPE groups using McNemar's test of related samples. There were statistically significant reductions in the proportions of participants with a PTSD diagnosis in the MPE and SPE groups from baseline (T1) to 12 months (T4). Neither group changed significantly from T3 to T4. At T4, 53.2% of the MPE group and 54.1% of the SPE group had no PTSD diagnosis; χ^2^(1, *n* = 134) = 0.002, *p* = 0.963.

The change scores for all secondary measures are presented in Table 2. Both treatment groups experienced reductions in anger, anxiety, and depression over time, and improvements in quality of life. Note that in Table 2 the difference score is an estimation of the change in the outcome measure over the two time points. A negative difference indicates that the score on the second occasion is lower than the score on the first occasion.

Neither group reported a significant deterioration in comorbid mental health issues. With the exception of disability scores for the MPE group, both groups improved significantly over time on all measures on at least one occasion. Small-to-moderate effects were found for change in anger, with moderate-to-large effects found for change in anxiety and depression scores. Finally, for both groups, there were no significant changes from 12 weeks to 12 months (T3 *v.* T4) for any of the outcome measures.

## Discussion

The current study aimed to assess whether the longevity of treatment gains for MPE therapy was non-inferior to SPE therapy, and to ascertain whether MPE was non-inferior to SPE in the impact on secondary outcome measures. As hypothesised, MPE and SPE maintained a reduction in PTSD severity at 12 months, consistent with outcomes reported at 12 weeks (Dell et al., 2022). Further, there were significant reductions in self-reported depression, anxiety, anger, and an increase in quality of life at the 12-week follow-up in both MPE and SPE groups, which were maintained at 12 months.

The emergence of MPE in recent years has been a promising advancement in the field, however until now, the long-term gains of a massed protocol have not been examined in a large-scale high-quality RCT. The effect size for PTSD symptom changes from baseline to 12 months was large and MPE was non-inferior to SPE. Further, the percentage of individuals who lost their diagnosis was sustained from T3 (Dell et al., 2022) to T4 in both groups. With massed protocols becoming increasingly population, it was critical to understand whether the process of fear consolidation and extinction could be learned and maintained over such a short duration. This RCT provides the first evidence to suggest that it can.

It is widely acknowledged that patients experiencing PTSD frequently have comorbid mental health issues, particularly military and veteran patients. While significant reductions in depression, anxiety, and anger have been reported following SPE therapy (Aderka et al., 2011; Eftekhari et al., 2013; Ford et al., 2018), the evidence for MPE has been less clear. Results of the current study are consistent with previous studies finding a reduction in anxiety and depression following MPE treatment (Hendriks, de Kleine, Broekman, Hendriks, & van Minnen, 2018; Zwetzig et al., 2022); however, the current study has also demonstrated for the first time, a reduction in anger symptoms following MPE therapy. From baseline to 12 weeks and 12 months, a small effect on anger was observed. Similar to previous studies, it is clear that residual anger can remain following trauma-focused treatment, including prolonged exposure and it may be that adjunct intervention needs to be considered to target anger more specifically (Ford et al., 2018; Zayfert & DeViva, 2004). Changes in anxiety and depression had moderate-to-large effect sizes, and importantly, reductions made in all comorbid mental health symptoms immediately after treatment were maintained at 12 months irrespective of the treatment condition.

Alongside the reductions in PTSD and other comorbid mental health conditions, improvements in quality of life were observed for both groups at 12 weeks and maintained at 12 months. This finding is consistent with recent SPE research (Schnurr et al., 2022) and a qualitative study (Sherrill et al., 2020); however it is novel in relation to MPE, where quality of life at 12 months post-treatment has not previously been reported in literature. This finding suggests a generalising of improvements more broadly into the individual's life. For the measure of disability, however, the only statistically meaningful change was observed from baseline to 12 weeks in the SPE group, a result not upheld at the 12-month follow-up. We propose that this may be due to several of the items on the disability measure (WHODAS) being specific to physical health ability (e.g. standing for long periods such as 30 min, walking a long distance such as a kilometre), none of which are directly addressed through prolonged exposure therapy. It may be that some of these items may are particularly difficult to change in military and veteran populations, where long-term physical injury and disability is a known risk and outcome of the occupation. Further research into the psychological symptoms contributing to self-reported disability, in particular, would allow for greater gains to be made around this factor.

The outcomes of this study should be considered with limitations in mind. First, measures of comorbid mental health conditions were all self-reported and may not accurately reflect the rates present in the population. Second, the extent to which these findings can be applied to populations other than military and veterans is yet to be determined, and with the sample being predominantly male, there is the question of applicability of the results to females. Third, the substantial observed loss to follow-up requires us to exert some caution in interpretation of the results. Lastly, while the current findings contribute to increasing evidence for MPE's clinical utility, it is worth acknowledging that prolonged exposure may be suitable for certain clinical presentations, while another evidence-based treatment may be most suitable for others. It is imperative characteristics relevant to optimal treatment response continue to be explored. Similarly, MPE may not be suitable for all therapists given the intensity of treatment delivery and requirement to allow for five sessions with one individual per week.

This study demonstrated that MPE was non-inferior to SPE in the reduction and maintenance of PTSD symptomatology and loss of diagnosis up to 12 months following therapy. Comparable reductions in depression, anxiety, and anger symptoms were also evident across the two treatments. The PTSD treatment field is cognisant of the front-line treatments for the disorder, however in a practical clinical setting, these treatments need to be offered in a modality that will suit patients and the demands of their life, particularly given that patients who are provided their preferred treatment are more likely to adhere and report greater reductions in self-reported mental health issues (Zoellner, Roy-Byrne, Mavissakalian, & Feeny, 2019). Having the flexibility to offer modifications of evidenced-based treatment with demonstrated long-term utility is a significant advancement in the field. Future research should explore predictors of treatment response to achieve a better understanding of the characteristics contributing to beneficial outcomes in MPE therapy as compared to SPE.
